# RNAprecis: Prediction of full-detail RNA conformation from the experimentally best- observed sparse parameters

**DOI:** 10.1063/4.0000988

**Published:** 2025-10-27

**Authors:** Christopher J Williams, Henrik Wiechers, Benjamin Eltzner, Jane S Richardson, Stephan F Huckemann

**Affiliations:** 1 Duke University, Durham, NC, USA; 2 University of Göttingen, Göttingen, Lower Saxony, Germany

## Abstract

RNA model building is particularly challenging at resolutions worse than about 2.5Å. The RNA backbone has a large number of degrees of freedom, but is frequently underdetermined due to ambiguous density.

Here we present RNAprecis, a structure validation system for predicting RNA backbone conformations at atomic detail from a set of minimal parameters which are reliably visible even in lower resolution maps. The work expands on our previous success in predicting RNA sugar puckers, features that are very difficult to see in density maps, from similar minimal parameters – a validation already available through MolProbity and Phenix. To overcome the large conformational space of RNA backbone, RNAprecis uses unsupervised machine learning on a curated dataset of high-quality, residue-filtered RNA models to cluster RNA backbone conformations. We test our predictive system on a set of RNA outlier conformations paired with related structures solved at higher resolution, showing good results, and some targeted challenges such as differentiating the 1a and 1c conformers.

As a bonus, the clustering algorithm has revealed at least one conformational cluster that was previously unrecognized, adding to our library of RNA conformers.

Preprint: https://www.biorxiv.org/content/10.1101/2025.02.06.636803v2

